# Off His Game

**Published:** 1897-06-26

**Authors:** 


### "Off His Game."

A particularly knowing sort of person has lately been instructing the public through the daily press as to the prevalence of certain new, and strange, and anomalous conditions which are, as he fancies in his ignorance, quite unknown to the medical profession. These ailments are said to be chiefly met with among the "sporting set," and may be briefly summarised in the phrase "losing one's nerve." Sometimes without any apparent reason, sometimes after an accident, trivial or serious, the patient falls into a state of nervous enfeeblement which, although "not mentioned in medical books" and such as "would puzzle a physician to diagnose," is said to be sadly real to those who are so afflicted.

The description given of the complaint is true enough; but the suggestion that it is a thing not recognised or understood by the medical profession appears to be the outcome of a curiously limited acquaintance with the present position of medical science.

The victim is said to "shun the herd" like a wounded deer. He finds that, bold as he used to be, he can no longer do or dare. The hunting man finds that he turns sick and faint at the sight of a stiff fence which once would have filled him with joy. He is said to have "no bodily ailment" but simply to have "lost his nerve." But we could show scores of instances quite outside the sporting set in which exactly the same thing occurs; in which the clergyman, accustomed to rouse his hearers by the boldness of his utterances, is found trembling at the thought of entering the pulpit, and when he gets there pouring out platitudes, not from want of intellect but from want of nerve; of business men who would have revelled in a "crisis"—and would probably have made money thereby—hesitating and doubting and making false moves from sheer "funk," even when the market is as steady as old Time; and of surgeons accustomed to active operative work falling back on "safe" opinions and expectant treatment. But all this is no strange and unknown thing. It is but our old friend neurasthenia, which crops up in so many forms, and shows itself in so many disguises. Unfortunately when these things happen there is always at hand the knowing man, the clever fellow who thinks that "doctors don't know everything," and incontinently persuades the sick man that it is all liver, and that he had better take a pill, or urges him to travel, and thus to wear out still further his exhausted nervous system. But neurasthenia cannot be walked off, nor "biked" off, nor can it be dissipated by the fatigues and weariness of foreign travel. Nutrition is at fault in all these cases, and where travel and change of scene do good, as no doubt they do in many instances, the good effect is far more the result of the better digestion which is enjoyed, and the larger amount of food which is taken when men are away from home and removed from all their worries, than from any mental influence of mere change.

Needless to say, however, that in serious cases even the travelling is itself a worry, and is often a not inconsiderable strain upon the weakened physical powers. In such cases the irresponsible advice of the knowing friend, to run away and get a change, is worse than useless; what is wanted is a properly devised rest-cure carried out under proper medical supervision. Unfortunately the dictum that "doctors don't know everything" too often holds people back from seeking medical advice unless they feel a pain, or see a lump, or find themselves possessed by some obvious physical disorder. When Macbeth asked the question, "Canst thou not minister to a mind diseas'd?" he sowed a seed of doubt which has done infinite harm to endless generations. People refuse to recognize how physical is the basis of their mental life, and when the active mistress of a busy house gets into a "low way," or a sportsman "loses his nerve," or the golfer finds that he is "off his game," the last thing they think of is going to a doctor; for, as they say, there is nothing the matter with them. So they take the advice of their various friends and do all sorts of silly things, ending too often in alcoholism and all its associated depravity. The secret drinking so common among refined and educated women, and the haunting of bars and smoke rooms by men who "in their day" had been such clever fellows is to a large extent the outcome of ill-treated or untreated neurasthenia. These people do not at first drink for the pleasure of it, but to remove their sinking miseries, and because they feel that without a fillip they cannot get along. A wider recognition of the fact that loss of nerve means a physical disorder of the nervous system which requires proper medical and dietetic treatment would prevent a great deal of that chronic alcoholism which is the saddest end to a bright intellect, and is an end by far too common.

